# Visualization of plasmonic diffraction-guided carrier dynamics in silicon photodetectors

**DOI:** 10.1515/nanoph-2025-0418

**Published:** 2025-11-26

**Authors:** Soh Uenoyama, Yusuke Yoshizawa, Kazunori Tanaka, Hiroyasu Fujiwara, Atsushi Ono

**Affiliations:** Central Research Laboratory, Hamamatsu Photonics K.K., 5000 Hirakuchi, Hamana-Ward, Hamamatsu City 434-8601, Japan; Graduate School of Integrated Science and Technology, Shizuoka University, 3-5-1 Johoku, Chuo-Ward, Hamamatsu City 432-8561, Japan; Research Institute of Electronics, Shizuoka University, 3-5-1 Johoku, Chuo-Ward, Hamamatsu City 432-8011, Japan

**Keywords:** NIR photodetectors, plasmonic-diffraction, visualizing photo absorption pathway

## Abstract

Silicon-based photodetectors operating in the near-infrared (NIR) wavelength range (*λ* = 700–1,100 nm) are essential for applications such as light detection and ranging, facial recognition, and eye-tracking. However, silicon’s low absorption coefficient in this range limits photodetection efficiency. While recent advances in nano-diffraction structures have improved photo-absorption by increasing the effective absorption path, optimizing carrier dynamics remains challenging. In the NIR regime, photons penetrate deeply into the silicon substrate, making it critical to align the spatial distribution of photo-generated carriers with the charge collection regions. However, the angular and spatial behavior of carrier generation (CG) and collection under nano-diffraction structures remain underexplored. This study presents an analytical model that visualizes CG pathways and corresponding collection probabilities induced by plasmonic diffraction structures, providing insight into diffraction-driven CG in silicon. The model is experimentally validated through photocurrent responses in non-illuminated neighboring pixels, directly revealing plasmonic diffraction effects. The results show that diffraction enhances light absorption and enables visualization of the CG and collection pathways based on the diffraction angle. This approach enables the spatial overlap of CG and collection pathways, efficiently guiding incident photons to photosensitive regions. This framework offers a new strategy to enhance NIR photodetector performance through diffraction-guided light propagation and device-specific modeling.

## 1 Introduction

Silicon (Si)-based photodetectors operating in the near-infrared (NIR) wavelength range (*λ* = 700–1,100 nm) have attracted significant attention for applications such as light detection and ranging (LiDAR) [1], [2], [3], [4], [5], facial recognition [6], [7], eye-tracking [8], [9], and remote sensing [10]. Here, we follow the convention in Si-based photodetector research and refer to *λ* = 700–1,100 nm as the NIR region. However, an inherently low NIR absorption of Si significantly limits its photodetector performance. A common mitigation strategy is to increase the thickness of the Si epitaxial (epi) layer [11], [12]. Nevertheless, thick epilayers often cause optical cross-talk between adjacent pixels in imaging applications. Additionally, photons that are absorbed deeper in the substrate yield less efficient carrier collection because of increased recombination. Thus, visualizing the internal photoabsorption pathway and controlling the spatial overlap between the carrier generation (CG) route and high carrier collection probability (CCP) regions are critical for improving NIR photosensitivity while minimizing cross-talk.

Recent advances in nanophotonic diffraction approaches demonstrate that surface texturing, such as Si nanoholes [13], [14], [15], black silicon [16], [17], Si nanowires [18], perovskite textures [19], and Si pyramids [20], [21], can enhance absorption by extending the optical path in Si via internal scattering, even with thin epilayers. Among these, we previously demonstrated a plasmonic diffraction structure using a periodic metal nanograting that diffracts incident NIR light into Si at large angles [22], [23], [24], [25], [26].

While such plasmonic gratings increase CG by effectively extending the optical path of light within Si, the relationship between diffraction-induced CG and CCP has not yet been quantitatively analyzed. This theoretical gap limits the optimization of diffraction-based photodetectors for NIR photosensitivity, particularly in guiding photons efficiently to the photosensitive regions.

In conventional approaches, the external quantum efficiency (EQE) is typically estimated by combining numerically obtained CG distribution from optical simulations with CCP distribution from electrical device simulation, followed by full-area integration [27]. Although this approach is accurate, it does not provide insight into the angle-resolved behavior of the diffraction-induced generation process and is computationally expensive, making iterative design optimization impractical. Therefore, the challenge is not the absence of modeling frameworks, but rather the lack of a simplified and tractable analytical model that captures the diffraction-angle-dependent CG and its linkage to CCP without requiring full optical–electrical co-simulation.

**Figure 1:**
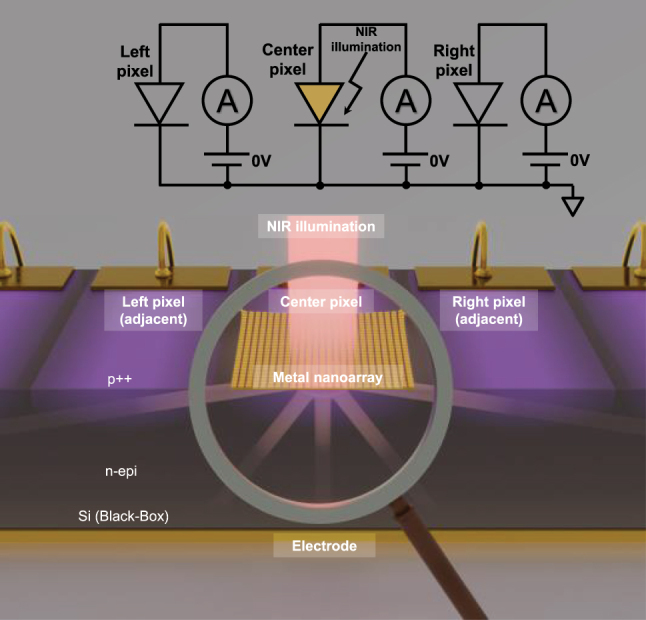
Conceptual illustration of plasmonic diffraction and the corresponding analytical model: a metal nanoarray is integrated on the center pixel of the 1D Si photodiode array, while the adjacent left and right pixels remain unpatterned and unilluminated. During measurement, the incident NIR light is focused only on the center pixel. The generated photocurrent is independently extracted from each pixel through a vertical p–n junction, where carriers are collected toward the bottom electrode. The diffracted light propagates laterally into the Si, where it induces CG in the adjacent pixels. The proposed analytical model enables visualization of the CG pathways and CCP inside the Si “black-box” region based on the plasmonic diffraction signals detected from adjacent pixels.

In this study, we develop a simplified analytical model that estimates the EQE by extracting the angle-resolved diffraction intensity from the near-field electrical field distribution of the nanograting, converting it into CG profiles using the Lambert–Beer law along the corresponding oblique optical paths, and integrating these with CCP obtained from device simulations. This approach enables intuitive understanding of the diffraction-driven carrier dynamics and significantly reduces computational cost compared with full optical–electrical co-simulations. To validate the model, we fabricate a gold (Au) nanoarray on a one-dimensional (1D) Si photodiode array. As a first step, we experimentally evaluate the photocurrent enhancement (PCE), defined as the ratio of photocurrent with and without the Au nanoarray, to observe the resonance wavelength of the plasmonic diffraction. Subsequently, by measuring photocurrents in adjacent, nonilluminated pixels, we experimentally observed plasmonic diffraction at a resonance wavelength and validated the model against the results. The model enables alignment of CG pathways with high-CCP regions (Figure 1) and supports the rational design of nanophotonics-enhanced Si photodiodes tailored for the NIR range.

## 2 Materials and methods

### 2.1 Device structure

**Figure 2:**
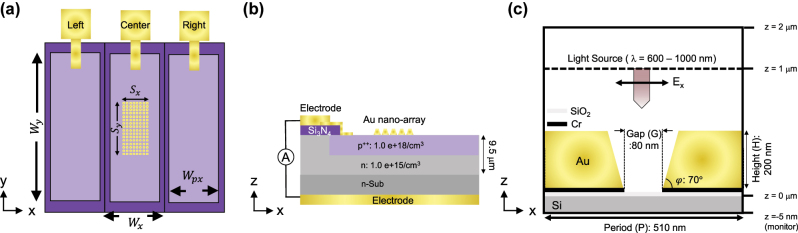
Schematic of the three-pixel segment of the 1D Si photodiode array. (a) Top view (*x*–*y* plane) showing the center pixel and its adjacent left and right pixels. Each pixel has lateral dimensions of *W*_*x*_ = 50 μm (*x*-direction) and *W*_*y*_ = 500 μm (*y*-direction). The Au nanoarray is patterned only on the center pixel, covering a region defined by *S*_*x*_ and *S*_*y*_, while the p++ carrier generation region has a lateral width of *W*_*px*_. (b) Cross-sectional view (*x*–*z* plane) illustrating the layer structure composed of a p++ region (1.0 × 10^18^ cm^−3^) formed within an *n*-type epilayer (1.0 × 10^15^ cm^−3^, thickness: 9.5 μm), grown on an *n*-type Si substrate (n-sub). A Si_3_N_4_ isolation layer and Ti/Au electrodes enable independent photocurrent extraction from each pixel via p++ contacts and the backside *n*-type electrode. (c) FDTD simulation model of the Au nanograting on a Si substrate. The model includes periodic Au grating with a period *P* = 510 nm, gap *G* = 60 nm, height *H* = 200 nm, and taper angle *φ* = 70°. A normally incident plane wave (*λ* = 1 µm) with *E*_*x*_-polarization was used for illumination. Detailed parameters are described in Section 2.4.

Figure 2a and b illustrate the device structure of the 1D Si photodiode array in the top view (*x*–*y* plane) and cross-sectional view (*x*–*z* plane), respectively. This study focuses on a three-pixel segment consisting of a center pixel and its adjacent left and right pixels. Each pixel has a lateral width of 50 μm in the *x*-direction (*W*_*x*_) and a length of 500 μm in the *y*-direction (*W*_*y*_). The device was formed on an *n*-type epilayer with a thickness of 9.5 μm and a doping concentration of 1.0 × 10^15^ cm^−3^, which was grown on an *n*-type Si substrate. A p++ region (doping concentration: 1.0 × 10^18^ cm^−3^) was formed within the epilayer via partial boron (B^+^) ion implantation, defining a carrier generation region with a lateral width of 40 μm in the *x*-direction (*W*_*px*_). The implantation depth profile is provided in Supporting Information S1. The Au nanoarray was patterned only on the center pixel and was electrically isolated from the electrodes. The device operates as a vertical p–n junction photodiode, with the top p++ region contacted by Ti/Au and the *n*-type substrate serving as the bottom electrode. The Au nanoarray-covered region had dimensions of 40 μm in width (*S*_*x*_) and 100 μm in length (*S_γ_*). A dielectric Si_3_N_4_ layer was deposited to prevent electrical crosstalk between the *p*-implanted region and the surrounding *n*-epilayer Ti/Au electrodes were formed on the p++ regions of the left, center, and right pixels, as well as on the backside *n*-substrate, enabling independent measurement of photocurrents from each pixel.

### 2.2 Fabrication procedure of the 1D photocetector

The 1D photodiode array was fabricated on an *n*-type epilayer (thickness: 9.5 μm, doping concentration: 1.0 × 10^15^ cm^−3^) grown on an *n*-type Si substrate. First, a p++ region (1.0 × 10^18^ cm^−3^) was formed at the center of each pixel by B^+^ ion implantation (the depth profile is provided in Supporting Information S1). Subsequently, a Si_3_N_4_ isolation layer was deposited to prevent electrical crosstalk between the *p*-implanted region and the surrounding *n*-epilayer. Ti (20 nm)/Au (200 nm) electrodes were then formed and partially attached to the p++ regions of each pixel, as well as to the backside substrate for *n*-type. an Au nanograting was patterned only on the center pixel by electron beam (EB) lithography and lift-off processing (Section 2.3). The whole procedure is provided in Supporting Information S2.

### 2.3 Fabrication procedure of the nanoarray

The 300 nm thick EB resist (ZEP520-A, ZEON Corp.) was spin coated on the Si substrate at 800 rpm/5 s, 6,500 rpm/60 s, and heated up to 180 °C for 3 min on a hot plate. Nanoarray patterns were EB irradiated and developed in the development solution (ZED-N50) for 2 min and post-baked up to 110 °C for 2 min. Chromium (Cr), for the adhesion layer, and Au were deposited via thermal evaporation through an EB resist pattern mask. The thicknesses of the Cr and Au layers were 1 and 200 nm, respectively. The sample was dipped in the solvent for 2 days to allow for lift-off. Finally, the Au nanoarray was patterned on the Si substrate.

### 2.4 2D FDTD simulation of the gold nanograting

Figure 2c shows the optical simulation model of the Au nanograting. Finite-difference time-domain (FDTD) simulations were performed using Lumerical FDTD solutions (Ansys Inc., Canonsburg, PA, USA). Periodic boundary conditions and perfectly matched layer (PML) were applied in the *x*- and *z*-axis, respectively. The simulation domain ranged from −255 nm to +255 nm in *x*-axis (total width: 510 nm) and from −5 nm to 2,000 nm in *z*-axis. The Au nanograting consisted of a period (*P*) of 510 nm, a gap (*G*) of 60 nm, a height (*H*) of 200 nm (from *z* = 3 nm–203 nm), and a taper angle (*φ*) of 70°, with a 2 nm-thick SiO_2_ layer and a 1 nm-thick Cr adhesion layer beneath the Au nanograting. A normally incident plane wave with wavelengths of 600–1,000 nm was illuminated from *z* = 1,000 nm under *E*_*x*_-polarized illumination (Additional simulations under oblique incidence are provided in the Supporting Information S3.), where the electric field is aligned parallel to the grating vector (*x*-direction), since this polarization efficiently excites diffraction. A near-field/transmittance monitor was placed at *z* = −5 nm to extract the electric field intensity, which was converted into far-field intensity to calculate the diffraction efficiency ratio (DER). A mesh size of 1 nm was applied within the region of −255 nm ≤ *x* ≤ +255 nm and 0 nm ≤ *z* ≤ 250 nm. Note that although the fabricated structure is a 2D Au nanoarray, under *E*_*x*_-polarized illumination the diffraction occurs primarily along the *x*-direction, effectively behaving as a 1D nanograting. Accordingly, the optical response obtained from the 2D FDTD model was used to parameterize the analytical mode in Section 3.1. The validity of approximating the nanoarray as a 1D grating under *E*_*x*_-polarization is confirmed by the 3D FDTD comparison shown in Supporting Information S4.

### 2.5 Numerical optimization of the nanograting period and geometry

We used FDTD and finite element method (FEM) simulations to investigate the EQE of photodiodes with Au nanograting. FDTD simulations provided a spatial CG distribution. The CCP distributions were obtained using FEM in Lumerical CHARGE solver, based on the electrical parameters of the device, such as the doping concentration and epilayer thickness. By overlapping and integrating these two distributions of CG and CCP, we obtained the photocurrent collection probability (in detail, see Supporting Information S5) [27]. Note that this calculation was performed using a single-period unit cell under periodic boundary conditions. This unit-cell-based approach enabled a significant reduction in computational cost compared with full-array simulations.

### 2.6 Experimental setup for characterizing the photocurrent

The experimental setup included a dark box. The input light (SuperK Fianium, NKT Photonics) was spectrally separated into individual wavelengths (*λ* = 600–1,000 nm) using a tunable filter (LTTF Contrast VIS HP8, NKT Photonics). The light was collimated using a pair of mirrors, passed through a linear polarizer and beam splitter, and focused onto the sample using an objective lens (M Plan Apo, 20×, NA = 0.4, Mitsutoyo, Japan). The reflected light was captured using a camera with a tube lens (*f* = 200 mm). The photocurrent generated by the photodetector during the wavelength sweep (*λ* = 600–1,000 nm, 2 nm steps) was measured and transmitted to a source meter (Model 2636B, Keithley). For imaging purposes, a white-light source was introduced along the same optical axis. Further details of the optical setup are provided in the Supporting Information S6.

## 3 Results

### 3.1 Analytical modeling of the angle-resolved photo absorption and carrier collection

**Figure 3:**
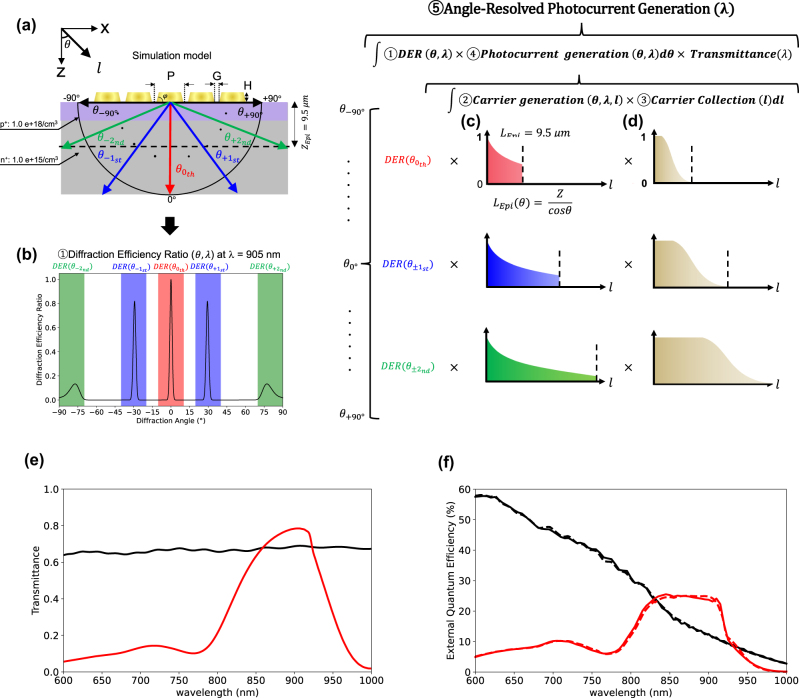
Analytical calculation procedure and simulation results: (a) schematic of the Au nanograting structure (the period (*P*), gap (*G*), height (*H*), and taper angle (*φ*). The epilayer thickness (*Z*_Epi_) is 9.5 μm. The *p*-type and *n*-type doping concentrations are 1.0e + 18/cm^3^ and 1.0e + 15/cm^3^, respectively. (b) DER as a function of diffraction angle at a wavelength of 905 nm. Red, blue, and green areas correspond to the 0th, 1st, and 2nd diffraction, respectively. (c) CG profile as a function of propagation length *l*. (d) CCP as a function of the effective propagation length *l*. (e) Transmittance spectrum as a function of the wavelength (*λ* = 600–1,000 nm) obtained from FDTD simulation. The red and black solid lines correspond to with and without the nanograting, respectively. (f) EQE plot as a function of the wavelength (*λ* = 600–1,000 nm). The red solid line and dashed line show that with the nanograting, and the black solid line and dashed line show that without the nanograting for numerical simulations and analytical calculations, respectively.

We theoretically developed an analytical model to visualize diffraction angle-dependent CG routes and their corresponding CCP induced by an Au nanograting (period (*P*) = 510 nm, gap (*G*) = 60 nm, taper angle (*φ*) = 70°, and height (*H*) = 200 nm), as illustrated in Figure 3a, optimized at a wavelength of 905 nm [26], typically used in LiDAR applications, through numerical simulation (see the Methods Section 3.4 for details). The analytical procedure involved the following steps (Steps 1–5). Figure 2a shows a schematic of this method.

Step 1: Diffraction Efficiency Ratio: DER (*θ*, *λ*)

The far-field intensity as a function of the diffraction angle was obtained by transforming the near-field intensity of the electric field, calculated using FDTD simulations, into the far-field domain.

Figure 3b shows the normalized DER, which represents the angular distribution of the far-field intensity converted from the near-field intensity of the nanograting at a wavelength of 905 nm, over a range of diffraction angles from −90° to +90°.

The Au nanograting supports multiple diffraction orders – specifically, the 0th, ±1st, and ±2nd orders – based on the incident wavelength and period of the grating. The corresponding diffraction angles are as follows:

(1) $$\theta =\text{si}{\text{n}}^{-1}\frac{m\lambda }{n_{\text{Si}}P}$$

where m denotes the diffraction order, *λ* is the incident wavelength, *n*_Si_ is the refractive index of the Si, and *P* is a period of the Au nanograting.

Step 2: Carrier Generation: CG (*θ*, *l*, *λ*)

The diffraction-angle-dependent normalized CG profile (Figure 2c) depends on the effective propagation length.

(2) $$l\left(\theta \right)=\frac{z}{\cos\theta }$$

where *θ* denotes the diffraction angle of the grating. Normalized CG is expressed as follows:

(3) $$\text{CG}\left(\theta ,l,\lambda \right)=\frac{I_{0}\exp\left(-\alpha l\left(\theta \right)\right)}{\overset{\infty }{\underset{0}{\int }}I_{0}\exp\left(-\alpha l\left(\theta \right)\right)\text{d}l}$$

where *I*_0_ and *α* are incident light intensity and absorption coefficient of the Si, respectively. As the diffraction angle *θ* increases (e.g., the diffraction order of 0th, 1st, and 2nd), the effective propagation length in Si substrate increases. Consequently, the CG becomes more spatially extended, eventually reaching the full thickness of the epilayer *Z*_Epi_ = 9.5 μm, as shown in Figure 3c. Note that all absorbed photons are assumed to be converted into CG.

Step 3: Carrier Collection Probability: CCP (*l*)

The spatial CCP was obtained using the FEM simulations, based on the doping concentration and thickness profile of the epilayer (see Supporting Information S1). The resulting CCP profile was obtained along the diffraction light propagation l-axis, as shown in Figure 3d.

Step 4: Photocurrent Generation: PG (*θ*, *l*)

To obtain the PG contribution at each diffraction angle, the CG and CCP were overlap integrated over the effective propagation route within the *n*-epilayer thickness along with l-axis, decomposed as *l* = (*l*_*x*_, *l*_*z*_)= (*l*sin*θ*, *l*cos*θ*).

Then the effective *n* epilayer thickness that depends on the diffraction angle is denoted as

(4) $$L_{\text{Epi}}\left(\theta \right)=\frac{Z_{\text{Epi}}}{\cos\theta }$$

PG is then given by:

(5) $$\text{PG}\left(\theta ,\lambda \right)=\overset{L_{\text{Epi}}}{\underset{0}{\int }}\text{CG}\left(\theta ,l,\lambda \right)\times \text{CCP}\left(l\right)\text{d}l$$

where

$$0\leq l_{z}\leq Z_{\text{Epi}}$$

This formulation accounts for both the spatial distribution of CG and the probability of CCP depending on the propagation path length determined by the diffraction angle.

Step 5: Angle-Resolved Photocurrent Generation: ARPG (*λ*)

To calculate the ARPG, the PG was integrated over all diffraction angles *θ* (ranging from −90° to +90°) of the product of DER and PG and then multiplied with the wavelength-dependent transmittance (*T*) of the nanograting (Figure 3e), where the black dash line and red solid line correspond cases with and without the nanograting:

(6) $$\text{ARPG}\left(\lambda \right)=T\left(\lambda \right)\overset{+90^{◦}}{\underset{-90^{◦}}{\int }}\text{DER}\left(\theta ,\lambda \right)\times \text{PG}\left(\theta ,\lambda \right)\text{d}\theta$$

Consequently, when multiplied by 100, this value corresponded to the EQE, expressed as a percentage:

(7) $$\text{EQE}\left(\text{\% }\right)=\text{ARPG}\left(\lambda \right)\times 100$$

Figure 3f plots the EQE spectrum obtained from the Au nanograting (period (*P*): 510 nm, height (*H*): 200 nm, gap (*G*): 60 nm, and taper angle (*θ*): 70°) calculated using both the analytical calculation and numerical simulation (see the Method Section 2.5). The black solid and dashed lines represent the EQE spectrum without the nanograting, obtained from numerical and analytical calculations, respectively. The red solid and dashed lines show the EQE spectrum with the nanograting based on analytical and numerical results, respectively. Consequently, the structure exhibits a distinct EQE peak at a wavelength of 905 nm. This strong agreement between the numerical simulation and the analytical model confirmed the validity of this model. The EQE enhancement observed at *λ* = 905 nm arises from multiple coupled effects. First, the high transmittance at this wavelength (Figure 3e) allows a large amount of light to enter the Si. Second, the diffraction occurs at a high angle of approximately 75°, which increases the effective optical path within the Si (Figure 3c). Third, this oblique propagation enhances the CCP efficiency by directing the carrier generation into regions with higher collection probability (Figure 3d). The combined contribution of these effects result in a pronounced EQE peak at *λ* = 905 nm. In contrast, at off-resonant wavelengths, the EQE is suppressed due to reduced transmittance and a weaker contribution from diffraction-enhanced carrier generation.

### 3.2 Fabrication results

**Figure 4:**
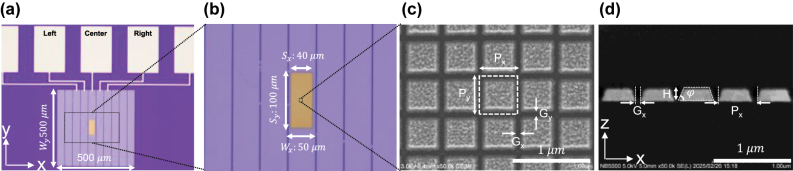
Fabrication results of the Au nanoarray: (a) optical microscope image of the Au nanoarray patterned on the 1D photodiodes. The total sizes of the 1D photodiodes are 500 μm × 500 μm. (b) The magnified view of (a). The dimensions of the Au nanoarray *S*_*x*_ are 40 μm in the *x*-direction, and *S*_*y*_ is 100 μm in the *y*-direction. The pixel sizes of the 1D photodiodes are 50 μm and 500 μm in the *x*- and *y*-directions, respectively. (c) SEM image showing a magnified view of (b). *P*_*x*_ and *P*_*y*_ denote the periods in the *x*- and *y*-directions, respectively; *G*_*x*_ and *G*_*y*_ represent the corresponding gap distances. (d) Cross-sectional SEM image of the nanograting structure. *P*_*x*_ denotes the period, *G*_*x*_ is the gap, *H* is the height, and *φ* is the taper angle of the nanoarray sidewall. The white scale bar is 1 μm.

Figure 4a shows a microscopic image of the Au nanoarray on a 10 × 1-pixel 1D arrayed Si photodiode. In this study, only three pixels (Center and its left and right neighbors) were used. The total size of the photodiodes was 500 μm × 500 μm. Figure 4b shows a magnified view of Figure 4a, confirming the pixel dimensions (*W*_*x*_ = 50 μm, *W*_*y*_ = 500 μm) and the Au nanoarray patterning area (*S*_*x*_ = 40 μm, *S*_*y*_ = 100 μm) are consistent with the designed structure as described in Section 2.1. Figure 4c shows the scanning electron microscopy (SEM) images of the Au nanoarray. The Au nanoarray was successfully fabricated. The period (*P*) and gap (*G*) of the Au nanoarray were 510 and 60 nm, respectively. To investigate the height and taper angle of the Au nanoarray, its side view was examined, as shown in Figure 4d. The taper angle (*φ*) and height (*H*) of the Au nanoarray were 70° and 200 nm, respectively.

### 3.3 Photocurrent enhancement (PCE) spectrum depending on the incident polarization

**Figure 5:**
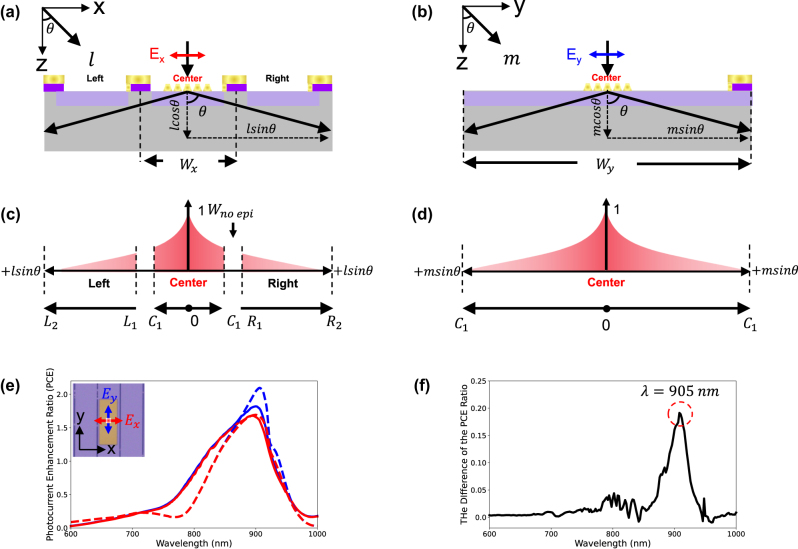
Photocurrent enhancement (PCE) ratio under *x*- and *y*-polarized illumination. (a, b) Schematic diagrams of the illumination configuration for the Au nanoarray under *x*-polarization and *y*-polarization, respectively. (c, d) Schematic illustration of the CG profiles as a function of *l*cos*θ* and *m*cos*θ* for *x*- and *y*-polarizations, respectively. (e) PCE spectrum as a function of wavelength (*λ* = 600–1,000 nm). Blue dashed and solid lines represent analytical calculation and experimental results under *y*-polarization, respectively. Red dashed and solid lines correspond to *x*-polarization. Inset figures show that magnified view, red and blue arrows correspond to the *x*- and *y*-polarization, respectively. (f) PCE difference between *x*- and *y*-polarizations plotted as a function of incident wavelength (*λ* = 600–1,000 nm).

To investigate the resonance wavelength of the plasmonic diffraction, we measured the photocurrent at the center pixel depending on the illumination polarization. Figure 5a and b shows the illumination of the Au nanoarray for *x*-polarization (*x*–*z* plane) and *y*-polarization (*y*–*z* plane), respectively. The analytical model should be modified in the regions of CG and CCP, which are redefined via incident polarization as follows (Figure 5c and d):

Center Region for *x*-polarization (*x*–*z* plane):

In the *x*–*z* plane, *l* = (*l*_*x*_, *l*_*z*_) = (*l*sin*θ*, *l*cos*θ*) and *θ* is the diffraction angle.

(8) $$\text{PG}\left(\theta ,\lambda \right)=\overset{L_{\text{C}1}}{\underset{0}{\int }}\text{CG}\left(\theta ,l,\lambda \right)\times \text{CCP}\left(l\right)\text{d}l$$

(9) $$L_{\text{C}1}=\frac{\text{C}1}{\sin\theta }$$

where

$$0\leq l_{x}\leq \text{C}1,\text{with}$$

(10) $$\text{C}1=\frac{W_{x}}{2}-\frac{W_{\text{no}\;\text{Epi}}}{2}$$

$$0\leq l_{\text{z}}\leq L_{\text{Epi}}$$

where the *W*_*x*_ is 50 μm for under *x*-polarization. The width of no epilayer *W*_no-epi_ is 10 μm, and C1 represents the distance from the center to the boundary between the center and adjacent pixels (left and right).

Center Region for *y*-polarization (*y*–*z* plane):

In the *y*–*z* plane, we defined *m* = (*m*_*y*_, *m*_*z*_) = (*m*sin*θ*, *m*cos*θ*), and *θ* is the diffraction angle.

(11) $$\text{PG}\left(\theta ,\lambda \right)=\overset{M_{\text{C}1}}{\underset{0}{\int }}\text{CG}\left(\theta ,m,\lambda \right)\times \text{CCP}\left(m\right)\text{d}m$$

(12) $$M_{\text{C}1}=\frac{\text{C}1}{\sin\theta }$$

where

$$0\leq m_{x}\leq \text{C}1$$

(13) $$\text{C}1=\frac{W_{y}}{2}$$

$$0\leq m_{\text{z}}\leq L_{\text{Epi}}$$

where the *W*_*y*_ is 500 μm under *y*-polarization.

We evaluated the PCE spectrum as a function of the incident wavelength (*λ* = 600–1,000 nm) for under *x*- and *y*-polarizations. The PCE is defined by the following equation:

(14) $$\text{PCE}\left(\lambda \right)=\frac{I_{\text{signal}}\left(\lambda \right)-I_{\text{dark}}\left(\lambda \right)}{I_{\text{ref}}\left(\lambda \right)-I_{\text{dark}}\left(\lambda \right)}$$

where *I*_signal_ and *I*_ref_ denote the photocurrents with and without the Au nanoarray, respectively. *I*_dark_ denotes the dark current of the photodiodes.

Figure 5e plots the PCE spectrum as a function of the incident wavelength. The red and blue solid lines are the experimental PCE spectrum, while the dashed line indicates the analytical simulation spectrum. These experimental results align with analytical calculation results. Additionally, Figure 5f shows the difference in the experimental PCE spectrum between the *x*- and *y*-polarizations. These polarization-dependent PCE differences pronounce that the plasmonic diffraction phenomenon occurred at a wavelength of 905 nm.

### 3.4 Photocurrent of the center and adjacent pixels via polarized illumination

**Figure 6:**
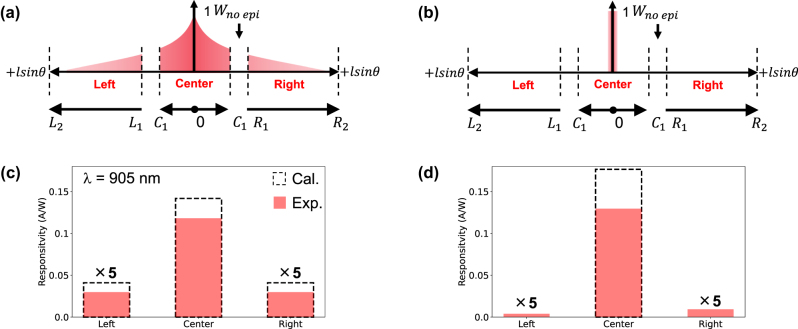
Spatial profiles of CG and collection across the electrode area under *x*- and *y*-polarized illumination: (a, b) schematic configurations illustrating the photoabsorption profiles under *x*-polarized and *y*-polarized illumination, respectively. (c, d) Photo responsivity values measured at the left (5×), center, and right (5×) pixels under *x*-polarization and *y*-polarization, respectively. Red bars and black-dashed outlined bars show experiment values and analytical calculations, respectively. For visibility, the left and right traces are multiplied by 5; actual values are one-fifth of the plotted height.

Subsequently, we measured the photocurrent at the center pixel and at adjacent pixels (left and right) to directly observe the plasmonic diffraction phenomenon at *λ* = 905 nm and validate the analytical model. To accurately model the PG, the photocurrent collection region was classified into center and adjacent (right/left) regions. The spatial profiles of CG and CCP across the electrode area under *x*- and *y*-polarized illumination are shown in Figure 6a and b, respectively. The integration domains for the photocurrent collection under *x*-polarized illumination are defined as follows:

Center region:

(15) $$\text{PG}\left(\theta ,\lambda \right)=\overset{L_{\text{C}1}}{\underset{0}{\int }}\text{CG}\left(\theta ,l,\lambda \right)\times \text{CCP}\left(l\right)\text{d}l$$

(16) $$L_{\text{C}1}=\frac{\text{C}1}{\sin\theta }$$

where

$$0\leq l_{x}\leq \text{C}1$$

(17) $$\text{C}1=\frac{W_{x}}{2}-\frac{W_{\text{no}\;\text{Epi}}}{2}$$

Adjacent pixel region (left/right):

(18) $$\text{PG}\left(\theta ,\lambda \right)=\overset{L_{\text{L}2},L_{\text{R}2}}{\underset{L_{\text{L}1},L_{\text{R}1}}{\int }}\text{CG}\left(\theta ,l,\lambda \right)\times \text{CCP}\left(l\right)\text{d}l$$

(19) $$\begin{array}{c} L_{\text{L}1}=\frac{\text{L}1}{\sin\theta },{\;L}_{\text{L}2}=\frac{\text{L}2}{\sin\theta }\\ R_{\text{L}1}=\frac{\text{R}1}{\sin\theta },{\;R}_{\text{L}2}=\frac{\text{R}2}{\sin\theta } \end{array}$$

with the integration bound defined as

$$\text{L}1,\text{R}1\leq l_{x}\leq \text{L}2,\text{R}2$$

(20) $$\text{L}1,\text{R}1=\left(\frac{W_{x}}{2}+\frac{W_{\text{no}\;\text{Epi}}}{2}\right)$$

(21) $$\text{L}2,\text{R}2=\left(\frac{3W_{x}}{2}-\frac{W_{\text{no}\;\text{Epi}}}{2}\right)$$

where L1 and R1 denote the edges of the photoactive area on the left and right sides, respectively, which are closest to the center pixel. L2 and R2 represent the edges of the photoactive area on the left and right sides, respectively, which are farthest from the center pixel. In these defined regions, particularly where no epilayer exists, CCP (*l*) is defined as zero owing to the absence of CG.

Figure 6c and d shows that the photocurrent values are measured at a center, left, and right pixel when the incident light is focused on the center of the Au nanoarray under *x*- and *y*-polarization, respectively. The responsivity was calculated by first determining the incident optical power using a calibrated reference photodiode (S2386-44K, Hamamatsu Photonics K.K.) under the same optical setup, and then dividing the measured photocurrent by the corresponding power. The detailed procedure and extracted values are provided in the Supporting Information S7. The red bars and the black-dashed outlined bars represent the experimental and analytical responsivity values, respectively, at a wavelength of 905 nm. Notably, we defined the illuminating center portion of the pixel to be the center of the Au nanoarray.

Under *x*-polarized illumination, a clear photocurrent is observed in adjacent pixels, indicating that the plasmonic nanoarray diffracts light laterally and symmetrically. In contrast, under *y*-polarized illumination, the photocurrent from adjacent pixels is negligible. These results demonstrate that plasmonic diffraction heavily depends on the polarization of the incident light and is consistent with the directional diffraction behavior of the nanoarray. Moreover, the measured responsivity values show good agreement with the analytical calculations.

### 3.5 Photocurrent of the center and adjacent pixel depending on incident light location

**Figure 7:**
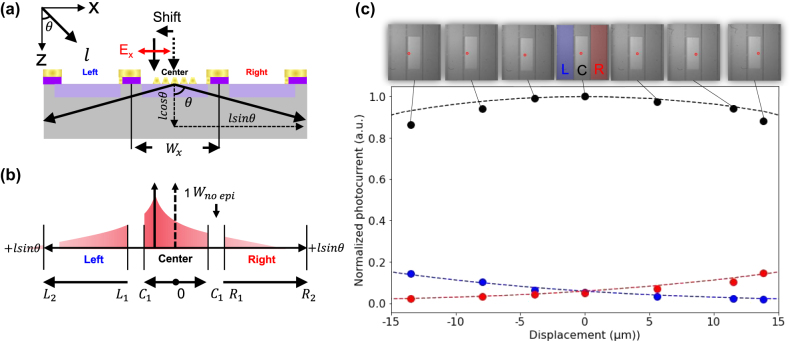
Photocurrent of the center and adjacent pixels depending on the illumination position: (a) the schematic illustration of the illumination configuration. (b) CG profile model, (c) normalized photocurrent as a function of the scanning illumination point. The black dot and dashed line correspond to the photocurrent for the center experiment and analytical calculation, respectively. The blue dot and dashed line correspond to the photocurrent for the left experiment and analytical calculation, respectively. The red dot and dashed line correspond to the photocurrent for the right experiment and analytical calculation, respectively.

To further validate the analytical model, we investigated the lateral shift of the incident light position in the PG under *x*-polarization (Figure 7a). The analytical model was modified to include a lateral shift in the illumination position (Figure 7b). In this extension, the integration bounds in the equations are updated to include a shift term.

Center region:

(22) $$\text{C}1=\frac{W_{x}}{2}-\frac{W_{\text{no}\;\text{Epi}}}{2}\pm \text{Shift}$$

Adjacent pixel region (left/right):

(23) $$\text{L}1,\text{R}1=\left(\frac{W_{x}}{2}+\frac{W_{\text{no}\;\text{Epi}}}{2}\right)\pm \text{Shift}$$

(24) $$\text{L}2,\text{R}2=\left(\frac{3W_{x}}{2}-\frac{W_{\text{no}\;\text{Epi}}}{2}\right)\pm \text{Shift}$$

The “+” and “−” signs indicate the direction of lateral displacement with respect to the adjacent pixel: a “+” denotes movement toward the adjacent pixel from the center, while a “−” denotes movement away from it.

Figure 7c shows the normalized photocurrents at the center, left, and right pixels as a function of the lateral displacement of the illumination spot. Normalization is defined as the maximum photocurrent of the experimental and analytical values at the center pixel. The black, red, and blue dots represent the experimental data, and the corresponding lines indicate the analytical results. These experimental results align with the analytical calculations, demonstrating that the analytical model accurately reflects the spatial dependence of PG with respect to the illumination position.

## 4 Discussion

In the analytical calculations, the Au nanograting supports multiple diffraction orders. The diffraction angle for each order was determined based on the period of the nanograting. When the period of the nanograting was 510 nm, the diffraction angles for the 0th, 1st, and 2nd diffraction orders were calculated to be 0°, 29.5°, and 80.3°, respectively, at a wavelength of 905 nm. The DERs across the diffraction orders were 30 %, 40 %, and 30 % for the 0th, 1st, and 2nd orders, respectively. In contrast, without the nanograting, only 0th-order diffraction was observed (see Supporting Information S8).

Despite the presence of small-angle 1st-order diffraction, the maximum PCE was obtained at 1.8 at a wavelength of 905 nm under *y*-polarization. This enhancement exceeded the transmittance difference ratio of 1.14 obtained from numerical simulations (see Section 3.1), which compared the transmission with and without the nanograting (78.4 % and 69.0 %, respectively). This discrepancy indirectly supports the contribution of plasmonic diffraction to PCE. Owing to fabrication limitations, we utilized a grating with a 2nd-order diffraction configuration (*P* = 510 nm). In future work, reducing the grating period by half to 255 nm would allow only 1st-order diffraction at large angles, which could significantly enhance PG at a wavelength of 905 nm.

Under *x*-polarized illumination with a pixel width of 50 μm, unabsorbed photons were diffracted from the center pixel to adjacent pixels (left and right). However, under *y*-polarized illumination with a large pixel width of 500 μm, almost all photons (95 %) were absorbed within the center pixel owing to the absorption length of Si being only ∼100 μm at a wavelength of 905 nm. Consequently, the PCE spectrum peak under *y*-polarization (1.82×) was slightly higher than that under *x*-polarization (1.68×). The difference in PCE between the polarizations clearly indicates that plasmonic diffraction under *x*-polarization allows significant diffraction of light leakage into adjacent pixels at a wavelength of 905 nm.

In the plasmonic diffraction observation experiment at a wavelength of 905 nm, a slight photocurrent was still observed in the adjacent pixel under *y*-polarized illumination. This residual signal is likely because of weak diffraction coupling along the *y*-direction, which can occur even under *x*-polarized illumination, since the 2D array geometry inherently supports multiple diffraction pathways. To further suppress the diffraction along the *x*-direction, we replaced the 2D array with a 1D grating structure aligned along the *x*-axis (see Supporting Information S4).

This structural modification effectively eliminated *x*-direction diffraction under *y*-polarized illumination, thereby confirming that the 1D grating geometry enhanced the polarization selectivity and enabled the directional control of diffraction-induced PG. This is particularly beneficial for active sensing applications such as LiDAR because it provides polarization filtering while simultaneously improving the photodetection performance and enhancing the signal-to-noise ratio.

This demonstration was conducted under focused illumination (NA = 0.4) as described in Section 2.6. To evaluate the angle dependence of the diffraction response, we calculated the transmittance as a function of incident angle using FDTD simulations (Supporting Information S3). Although plasmonic resonances are often highly angle-sensitive, the resonance in the present nanoarray is governed by collective localized surface plasmon resonances (LSPRs) rather than propagating surface plasmon polariton (SPP) modes. Owing to the weak angular dispersion of these collective LSPR modes, the coupling efficiency remains high over a range of incident angles [26]. Consequently, the Au nanograting retains approximately 70 % of its normal-incidence transmittance within ±25°, which corresponds to the angular range covered by NA = 0.4, indicating that the diffraction-induced response is robust under practical illumination conditions.

The sensitivities measured at each pixel (center, left, and right) as a function of the lateral displacement of the scan point were in close agreement with that of the analytical model for all scanned positions. In practical applications where illumination typically covers the entire dimensions of an individual pixel, this model is validated across all scanned points. Thus, the proposed analytical model is not limited to center illumination but can be reliably extended to spatially distributed illumination within individual pixels, ensuring its applicability to more finely structured pixel configurations.

The numerical simulation results combining the FDTD and FEM simulation results aligned with the proposed analytical calculations. These results indicate that the proposed model is highly accurate. Moreover, its calculation significantly reduces the computational memory consumption and processing time compared with full-wave numerical methods such as FDTD simulations. Specifically, the analytical method requires only 7.2 GB of memory, whereas the FDTD simulation consumes approximately 2.6 TB. Conventional FDTD approaches typically require modeling the entire Si region, including the gold nanograting – for example, spanning 3 pixels along the *x*-axis (150 μm) and covering an epilayer thickness of 9.5 μm along the *z*-axis – resulting in substantial computational overhead. In contrast, our analytical approach enables rapid dataset generation, facilitating a more efficient device structure optimization.

Furthermore, our analytical model visualizes the PG regions and elucidates the pathways of PG within the Si substrate via diffraction angles. This capability enables precise adjustment of trench positioning in imaging applications and fine-tuning of the doping concentration and thickness of the epilayer to achieve spatial alignment between the CG and CCP regions.

## 5 Conclusions

We developed an analytical model to visualize the unobservable CG route and its corresponding CCP within Si via diffraction angles. To validate this approach, we fabricated the Au nanoarray on 1D photodiodes and experimentally observed the plasmonic diffraction phenomena by measuring the photocurrent in the center and adjacent pixels that were not directly illuminated. The experimental results showed good agreement with the analytical predictions, demonstrating the validity of our model. Furthermore, the analytical model was extended to account for lateral variations in the incident light position, and the corresponding experimental results showed a strong correlation. These findings demonstrate that the internal light propagation route in Si, which is traditionally regarded as a “black box,” can be visualized through diffraction-induced lateral PG. The proposed method can optimize photodetector configurations, such as trench depth, geometry, epilayer thickness, and concentration. This study establishes a new strategy for designing photodetectors optimized specifically for nanophotonic light manipulation via diffraction.

## Supplementary Material

Supplementary Material Details
