# Supplementary material for: Visualization of plasmonic diffraction-guided carrier dynamics in silicon photodetectors
Source: Nanophotonics. 2025 Nov 26;14(25):4615–26. doi: 10.1515/nanoph-2025-0418 (PMC12714044; doi:10.1515/nanoph-2025-0418)
Supplement: Supplementary file 1 — Supplementary Material Details [file j_nanoph-2025-0418_suppl_001.pdf]

Supporting Information

Visualization of Plasmonic Diffraction-Guided Carrier Dynamics in Silicon Photodetectors

Soh Uenoyama, Yusuke Yoshizawa, Kazunori Tanaka, Hiroyasu Fujiwara, and Atsushi Ono*

Soh Uenoyama*, Yusuke Yoshizawa, Kazunori Tanaka, Hiroyasu Fujiwara,

Central Research Laboratory, Hamamatsu Photonics K.K., 5000 Hirakuchi, Hamana-ward, Hamamatsu City 434-8601, Japan

E-mail: so.uenoyama@crl.hpk.co.jp

Atsushi Ono 2,3

2: Graduate School of Integrated Science and Technology, Shizuoka University, 3-5-1 Johoku, Chuo-ward, Hamamatsu City 432-8561, Japan

3: Research Institute of Electronics, Shizuoka University, 3-5-1 Johoku, Chuo-ward, Hamamatsu City 432-8011, Japan

S1: Ion Implantation and SIMS Profile

Figure S1a shows the cross-sectional structure of the photodiode. The p-type region was formed by boron (B^+) ion implantation, with a peak concentration of approximately 1×10^{18} cm^{-3} and a junction depth of ~250 nm. Figure S1b presents the secondary ion mass spectrometry (SIMS) profile of the B^+ distribution within the n-type Si epilayer (n-epi). The depth in the SIMS profile is defined with the p-layer surface as the origin (z = 0 nm).

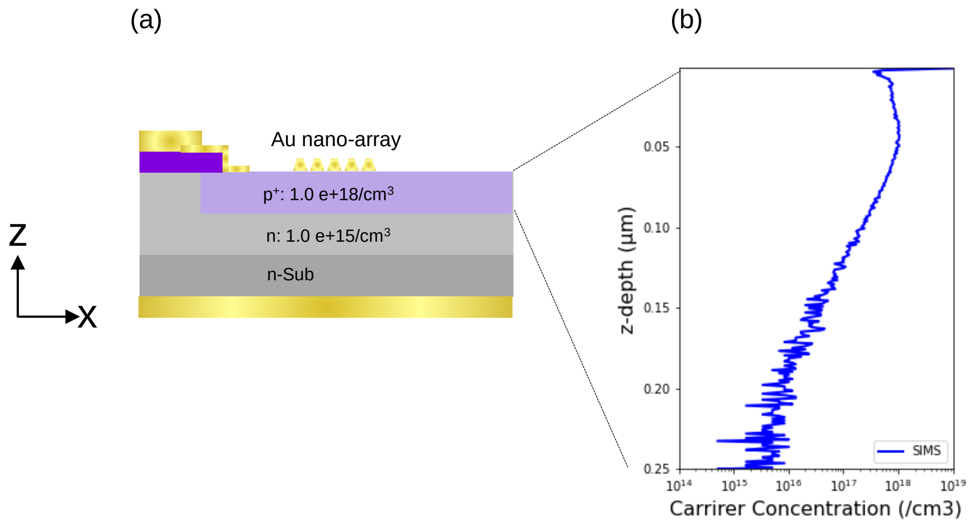

Figure S1: Fabrication procedure of the photodiodes

(a) Schematic of the illumination. (b) SIMS profile, carrier concentration plot ($/cm^3$) as a function of the z-depth.

S2: Fabrication procedure of the Au nanoarray on the photodiode

Figure S2 shows the fabrication procedure of the nanoarray on the photodiode as discribed in Section 2.2 and 2.3.

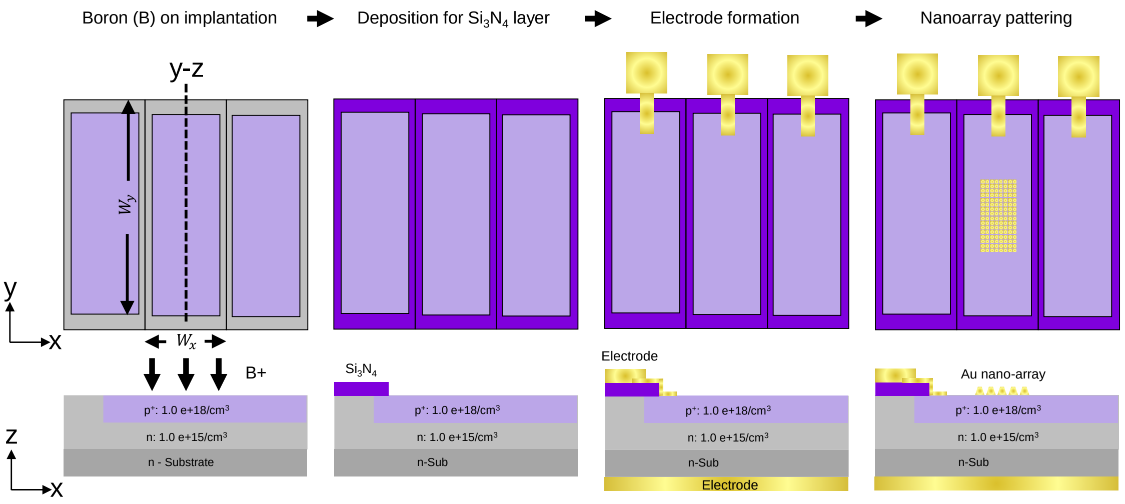

Figure S2: Fabrication procedure of the Au nanoarray on the 1D photodiodes.

S3: Incident angle-dependent transmittance of the Au nanograting

Figure S3a shows the transmittance spectra of the Au nanograting under E_x-polarized illumination as a function of wavelength for various incident angles (0° to 60°, in steps of 10°). Figure S3b presents the normalized transmittance as a function of the incident angle. The normalization is defined as the normal-incidence transmittance.

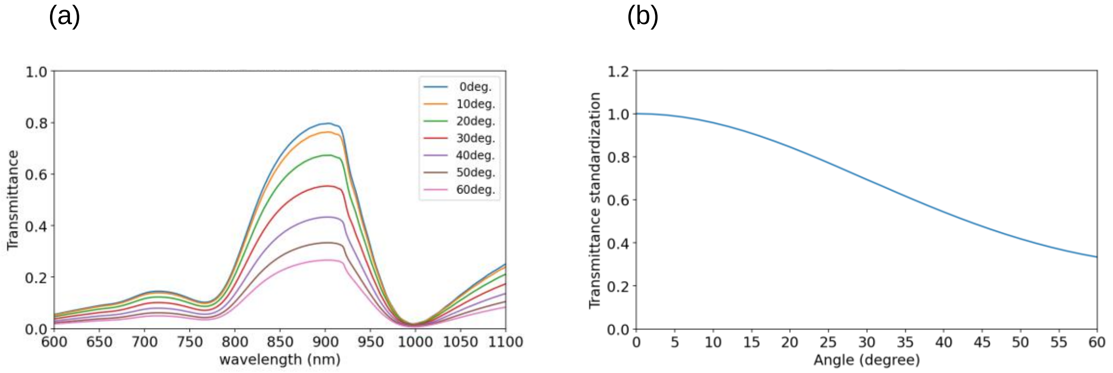

Figure S3: Incident angle–dependent transmittance of the Au nanograitng.

(a) Transmittance spectra of the Au nanograting under E_x-polarized illumination for various incident angles (0°–60° in 10° increments). (b) Normalized transmittance as a function of the incident angle, where normalization is performed with respect to the normal-incidence transmittance.

S4: Three-dimensional simulation model and far-field intensity map

To verify the polarization-dependent diffraction characteristics of the nanoarray and to justify the use of a 1D grating approximation in the main analysis, 3D FDTD simulations were performed for both structures. Figures S4a and d show the 3D FDTD simulation models of the nanoarray ($P_x = P_y = 510$ nm, $G_x = G_y = 80$ nm, H = 200 nm) and the corresponding 1D nanograting ($P_x = P_y = 510$ nm, $G_x = 80$ nm, $G_y = 0$ nm, H = 200 nm), respectively.

Figures S4b and c display the far-field intensity maps $I(\theta_x,\theta_y)$ of the nanoarray under E_x- and E_y-polarized illumination, respectively, where the red and blue profiles correspond to angular distributions along θ_x and θ_y, respectively. In the nanoarray, diffraction occurs along the x-direction under E_x-polarized illumination and along the y-direction under E_y polarization due to its two-dimensional periodicity.

Figures S4e and f show the corresponding far-field intensity maps for the nanograting. In this case, diffraction is confined along θ_x, where the grating periodicity exists, while illumination polarized perpendicular to the grating vector (E_y polarization) results primarily in reflection rather than diffraction. Furthermore, under E_x polarization, the nanograting exhibits significantly weaker diffraction leakage in the y-direction than the nanoarray, indicating a strongly one-dimensional diffraction behavior.

In addition, under E_x-polarized illumination, the primary diffraction characteristics of the nanoarray closely resemble those of the 1D nanograting. Therefore, the use of a 2D FDTD grating model in the main analysis was validated, even though the fabricated structure is a two-dimensional nanoarray.

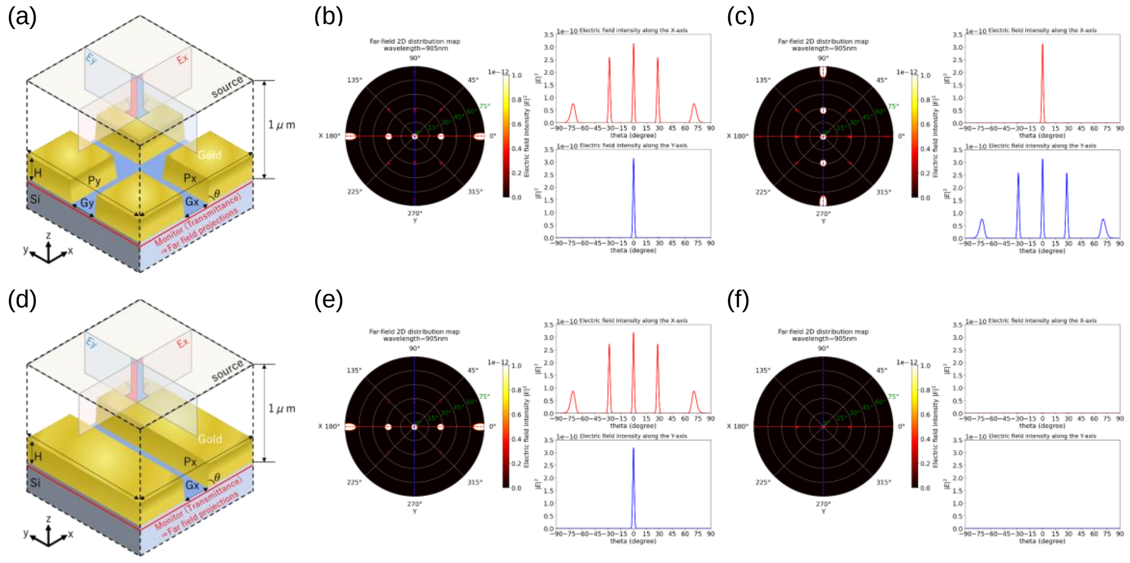

Figure S4: 3D FDTD simulations of the nanoarray and nanograting, and the corresponding far-field intensity maps and angular profiles.

(a, d) 3D simulation models of the nanoarray and nanograting, respectively. (b, c) Far-field intensity maps $I(\theta_x, \theta_y)$ of the nanoarray under E_x- and E_y-polarized illumination, respectively. (e, f) Corresponding far-field maps for the nanograting under E_x- and E_y-polarized illumination. The red and blue lines indicate the far-field intensity profiles along θ_x and θ_y, corresponding to E_x- and E_y-polarized excitation, respectively.

S5: CHARGE simulation configuration

Device simulations were performed to obtain the carrier collection probability (CCP) distribution using the Lumerical CHARGE solver. Figure S5a shows the electrode configuration used in the simulation, corresponding to the practical pixel arrangement consisting of the Center pixel and its adjacent pixels (Left1, Left2, Right1, and Right2). The pixel pitch was 50 μm, consistent with the fabricated device. The p++ region had a doping concentration of 1.0×10^{18} cm^{-3}, which matched the experimentally measured SIMS profile shown in Figure S1. The n-type epi layer had a doping concentration of 1.0×10^{15} cm^{-3} and a thickness of 9.5 μm.

Figure S5b shows the CCP map for one period of the Au nanograting, obtained from the CHARGE simulation. Figure S5c shows the carrier generation (CG) distribution for the same periodic unit cell, obtained from FDTD simulations. The EQE was estimated by overlap-integration of the CG distribution and the CCP map as follows [1]:

$$\mathrm{EQE}\ (\lambda) = \int \mathrm{CG}(x, y, \lambda)\mathrm{CCP}(x, y)dxdy$$

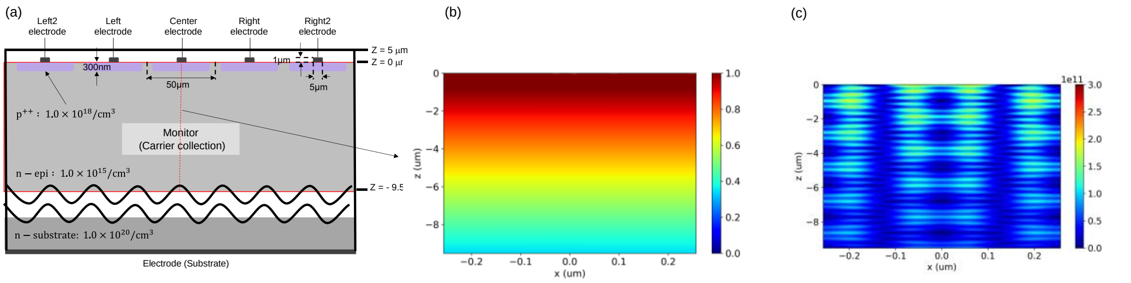

Figure S5: CHAERGE simulation configuration

(a) Electrode configuration used in the CHARGE simulation.

(b) Carrier collection probability (CCP) map for one grating period.

(c) Carrier generation (CG) distribution obtained from FDTD for the same periodic unit cell.

S6: Experimental Set-up

The experimental setup is shown in Figure S6.

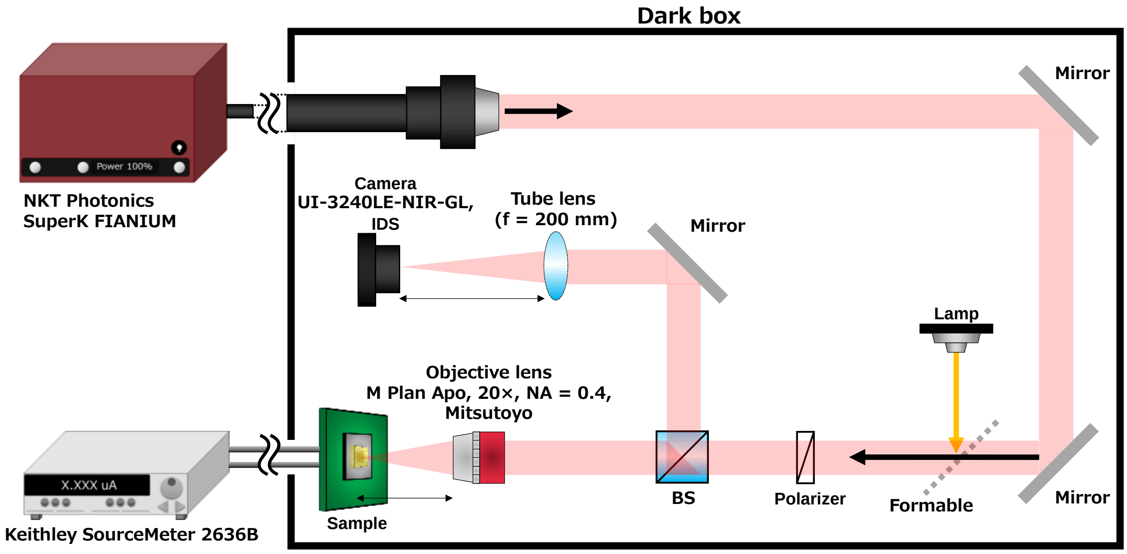

Figure S6: Experimental setup for characterizing the photocurrent of the sample. BS: beam splitter.

S7: Responsivity (A/W) calculation procedure

To obtain the responsivity (A/W) of the plasmonic photodetector, the incident optical power was first determined using a reference Si photodetector (S2386-44K, Hamamatsu Photonics K.K.). A schematic illustration of the experimental setup is shown in Figure S7a. Under the same illumination conditions as those used for the data in Figure 5(c,d) (main manuscript), with a spot size of approximately 5 μm in diameter, the photocurrent of the reference device was measured for both polarization states (Figure S7b). The incident optical power was calculated for each polarization, as summarized in Figure S7c using its known responsivity data,

Subsequently, the photocurrent generated by the plasmonic photodetector was measured under identical conditions. Although the structural pixel area is defined as W_{px}×Wy, the effective active area corresponds to the irradiated laser spot.

Therefore, the responsivity values shown in Figure 5(c,d) (main manuscript) were obtained by dividing the measured photocurrent by the corresponding incident optical power, assuming that the illuminated region constitutes the effective active area.

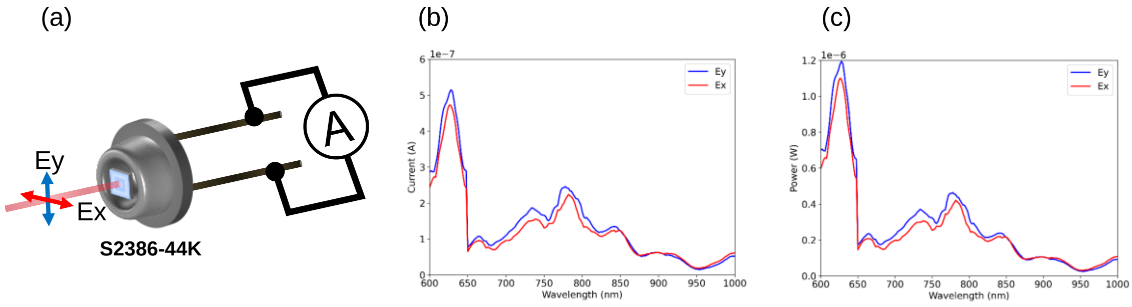

Figure S7: Responsivity calculation procedure

(a) Schematic illustration of the experimental setup.

(b) Measured photocurrent as a function of the incident wavelength.

(c) Light intensity as a function of the incident wavelength, where the red and blue plots correspond to Ex- and Ey-polarized illumination, respectively

S8: Far-field simulation result

The far-field intensity map is obtained as a function of the diffraction angle and incident wavelength by converting the near-field electric intensity obtained using finite-difference time-domain (FDTD) simulation to far-field light intensity. Figure S8a and b show schematic illustrations without and with the nanograting, respectively. Figure S8c and d show the far-field intensity maps with and without the gold nanograting as a function of the diffraction angle and incident wavelength, respectively. Without the nanograting, only 0th-order diffraction is observed, as shown in Figure S8e. In contrast, the gold nanograting supports multiple diffraction orders—0th, ±1st, and ±2nd—depending on the incident wavelength, as illustrated in Figure S8f. The diffraction efficiency ratios for the 0th-, ±1st-, and ±2nd-order diffractions are approximately 30%, 40%, and 30%, respectively. These values are calculated based on the angular dimensions of each diffraction order.

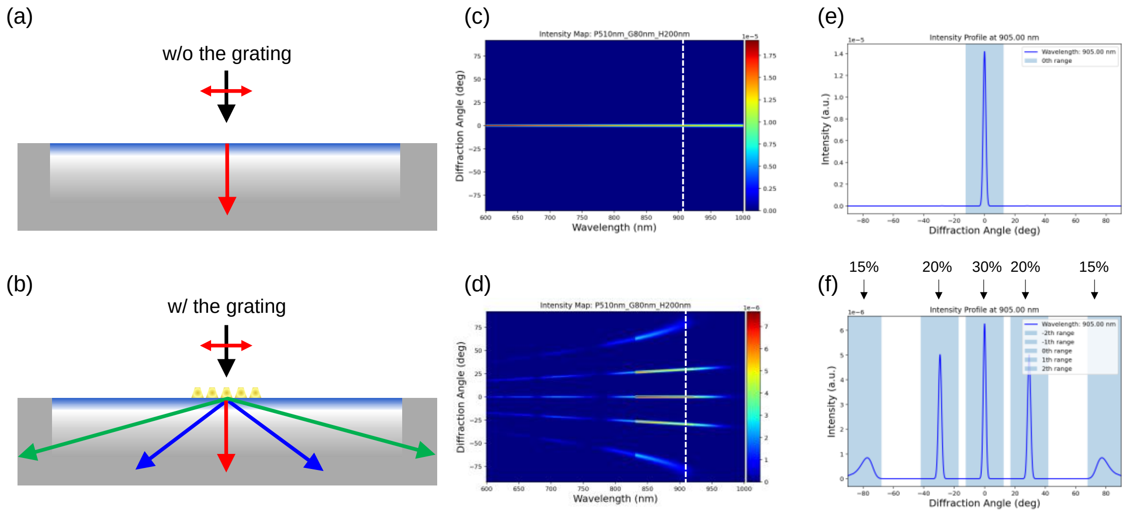

Figure S8: Far-field map and diffraction efficiency ratio

(a) Schematic of illumination without and with the nanoarray. (c) and (d) show the far-field light intensity as a function of the diffraction angle and incident wavelength. (e, f)

shows the diffraction efficiency ratio plot as a function of the diffraction angle at a wavelength of 905 nm with and without the nanoarray, respectively.

References

[1] E. Cobo et al., “Design of a CMOS image sensor pixel with embedded polysilicon nano-grating for near-infrared imaging enhancement,” Appl. Opt., vol. 61, no. 4, p. 960, 2022.
